# CubeSats deliver new insights into agricultural water use at daily and 3 m resolutions

**DOI:** 10.1038/s41598-021-91646-w

**Published:** 2021-06-09

**Authors:** Bruno Aragon, Matteo G. Ziliani, Rasmus Houborg, Trenton E. Franz, Matthew F. McCabe

**Affiliations:** 1 Water Desalination and Reuse Center, Division of Biological and Environmental Sciences and Engineering, King Abdullah University of Science and Technology, Thuwal, Saudi Arabia; 2 Planet, San Francisco, CA USA; 3 School of Natural Resources, University of Nebraska-Lincoln, Lincoln, NE USA

**Keywords:** Hydrology, Sustainability

## Abstract

Earth observation has traditionally required a compromise in data collection. That is, one could sense the Earth with high spatial resolution occasionally; or with lower spatial fidelity regularly. For many applications, both frequency and detail are required. Precision agriculture is one such example, with sub-10 m spatial, and daily or sub-daily retrieval representing a key goal. Towards this objective, we produced the first cloud-free 3 m daily evaporation product ever retrieved from space, leveraging recently launched nano-satellite constellations to showcase this emerging potential. Focusing on three agricultural fields located in Nebraska, USA, high-resolution crop water use estimates are delivered via CubeSat-based evaporation modeling. Results indicate good model agreement (r^2^ of 0.86–0.89; mean absolute error between 0.06 and 0.08 mm/h) when evaluated against corrected flux tower data. CubeSat technologies are revolutionizing Earth observation, delivering novel insights and new agricultural informatics that will enhance food and water security efforts, and enable rapid and informed in-field decision making.

## Introduction

Globally, agriculture accounts for 85% of consumptive water use (i.e., water that is not returned to terrestrial water systems)^1^. Furthermore, even though approximately 80% of arable area is rainfed^2^, irrigation accounts for 72% of fresh water withdrawals^3^, while delivering almost 40% of crop production^4^. Due in large part to a growing sectoral demand, along with increasing awareness of the risks to sustainable water supplies, there has been a push to deliver “more crop per drop” to satisfy both future food requirements and secure freshwater availability^5,6^. Securing hydric resources, mitigating scarcity, monitoring consumption, and enforcing water rights, all require accurate measurements of evaporation (E) at different spatiotemporal scales^7^, with one of the most common and cost-effective approaches to retrieve E being the use of satellite-based platforms^8^.

Over the last several decades, government and private space agencies have launched numerous satellite missions^9^, providing unprecedented volumes of information on the Earth’s surface based on an increasing range of sensing capabilities^8^. Indeed, one of the most important collections for Earth science has been the decades long Landsat missions, which is responsible in large part for the rapid development of remote sensing as a science^10^. However, traditional satellite missions pose a significant monetary burden, with a typical launch costing on the order of billions of dollars^11^. Using a range of these space-based platforms, there have been many studies that have investigated remotely sensed E, particularly at the regional-to-global scale^12–15^. While such efforts are essential to characterize and describe large-scale processes and behavior, they have been produced at coarse spatial resolutions (generally between 1 and 25 km), which precludes their use in capturing smaller scale patterns and variability. Indeed, many of these approaches do not explicitly account for agricultural systems: at least at the field- and farm-scales needed for operational management and agricultural insights. Such information is particularly important for applications at the precision agricultural scale^16^, where farm management strategies such as irrigation scheduling^17^ and nutrient management^18^ represent key control variables. A commonly used solution to cope with the spatiotemporal limitations of traditional satellite platforms is to use image or sensor fusion approaches^19^. For instance, Fisher, et al.^20^ combined Landsat-8 together with land surface temperature (LST) from the ECOsystem Spaceborne Thermal Radiometer Experiment on Space Station (ECOSTRESS) sensor to retrieve E at 70 m resolution every 5 days. In a high-resolution example, Guzinski and Nieto^21^ employed a machine-learning fusion framework to retrieve E at 10 m spatial resolution every five days, using data from the Sentinel-2 and Sentinel-3 satellites. A number of approaches have also explored the fusion of higher spatial resolution LandSat data with the enhanced temporal resolution of MODIS to develop a 30 m daily product^22,23^. Although these and related studies provide E at varying resolutions and scales, none have achieved the long-term high-spatial and high-temporal (daily) resolution retrievals needed to drive precision agricultural applications and advances^16,24^.

An alternative approach to single-mission driven Earth observation is one based instead on many nano-satellites (referred to herein as CubeSats) that are launched in constellations, which can act in unison to collect high-resolution data across the globe at near-daily scales^25^. CubeSats leverage sensor and electronics miniaturization, improvements in power supply and consumption, availability of off-the-shelf components, and reusable launch vehicles that offer launch costs that are a fraction of traditional satellite missions^26,27^. On the other hand, CubeSat sensors may lack the quality and rigorous calibration of more traditional research-grade sensors and incur cross-sensor discrepancies when acting as a constellation: although harmonization strategies have been developed to overcome these constraints^28,29^. Recently, Houborg and McCabe^30^ demonstrated the utility of CubeSats for precision agriculture, employing Planet imagery to produce 3 m resolution normalized difference vegetation index (NDVI) maps. Building on this work, McCabe, et al.^31^ showcased the potential of using CubeSat data for producing high-resolution vegetation and terrestrial evaporation, while Aragon, et al.^17^ demonstrated CubeSat capability using a Priestley-Taylor based evaporation model to predict E over irrigated farmland in Saudi Arabia. Although previous studies have highlighted the spatial resolution advantages of CubeSats, none have provided E at the temporal scales required to accurately capture and track in-field crop water use dynamics^32^ (i.e., daily) until now.

Here we present the first daily CubeSat-based retrievals of E at 3 m resolution, using a year-long record of Planet (www.planet.com) cloud-free surface reflectances to estimate crop water use over three agricultural sites in Nebraska, USA during the 2019 growing season. Our results detect heterogeneous plant growth and capture the day-to-day variability in E, providing new agro-informatic metrics to drive in-field management decisions^33^ and timely responses to changing crop conditions^34^. CubeSats overcome the spatiotemporal constraints inherent in traditional Earth observation, providing the information required to deliver the promise of precision agriculture^34^, and drive advances in crop modeling^35^, forecasting, and yield prediction^36^. Apart from showcasing paradigm changing advances in Earth observation, our work demonstrates the game-changing potential that CubeSats offer to a variety of fields, particularly those where space and time constraints have limited process insights and advances.

## Methods

### Meteorological and evaluation data

**Figure 1:**
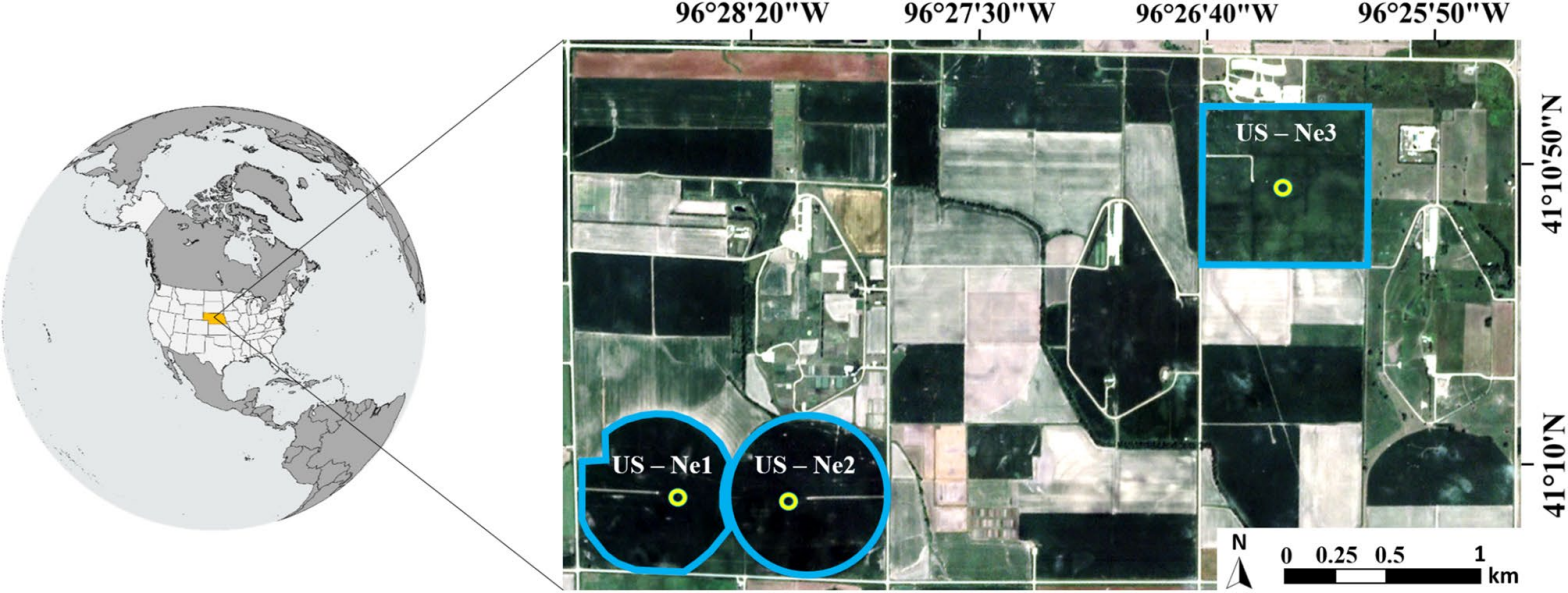
Location of the three field sites forming part of the University of Nebraska-Lincoln (UNL) Eastern Nebraska Research and Extension Center (ENREC) (see https://extension.unl.edu/statewide/enre/). The blue lines represent the approximate boundary of each field, while green circles indicate the location of eddy covariance towers in each field.

The study region is characterized by cold winters, hot summers, and overall humid conditions^37^ and has an extensive history of agronomic data collections and analysis^38^. Meteorological data used for model forcing, together with surface heat flux data that were used for evaluation, were sourced from eddy covariance (EC) towers installed at three different field sites (US-Ne1, US-Ne2, and US-Ne3) that are part of the University of Nebraska-Lincoln (UNL) Eastern Nebraska Research and Extension Center (ENREC) (Fig. 1). The flux towers collect data from both irrigated and rain fed crops (see Table 1), and contribute to the AmeriFlux network of eddy covariance stations (https://ameriflux.lbl.gov/). The meteorological data were recorded from the EC tower weather stations and provided hourly measurements of air temperature (*T*_*a*_, in °C) and relative humidity (*RH*, as a range between 0 and 100%). Needed hourly net radiation (*R*_*n*_) and latent heat flux (*LE*), sensible heat flux (*H*) and soil heat flux (*G*) were all measured in W/m^2^. EC towers are considered the gold standard for flux evaluation^39,40^, even though they commonly underestimate heat flux values and require an energy closure correction^38,41^. As there is no consensus on the most effective energy closure correction procedure^42^, we evaluate the modeled latent heat flux using the residual corrected *LE* fluxes (i.e., $LE = R_{n} - G - H$), which assumes that the H flux was measured correctly. Ten months of data were processed, with only daytime values used and records outside the crop growing season omitted for the evaluation process. Both meteorological and flux data were provided at hourly time steps for all days in 2019. Missing records, which affected around 1.4% of the EC data, were filled using simple linear interpolation. Latent heat fluxes that were negative during the day (representing approximately 2.5% of the records) were replaced by interpolated values on the assumption that condensation during daytime hours is unlikely^43^.

**Table 1:** Description of each study site, including the location, crop type, total rainfall, irrigation and fertilizer applications. These data are based on management logs taken during the course of the growing season.

Ameriflux ID	US-Ne1	US-Ne2	US-Ne3
Latitude, Longitude	41.1653, − 96.4766	41.164, − 96.4701	41.179, − 96.439
Crop type	Continuous maize	Rotation maize-soybean	Rotation maize-soybean
Sowing dates	April 19, 2019	April 23, 2019	April 24, 2019
Harvesting dates	November 5, 2019	November 7, 2019	November 7, 2019
Fertilizer (kg of N as urea/ha)	175 on April 16, 2019	157 on April 15, 2019	157 on April 15, 2019
Fertigation (kg of N as urea/ha)	45 on July 1, 2019	45 on July 2, 2019	N/A
Irrigation Type	Automatic with 90% efficiency		Rainfed
Irrigation Events (mm)	6.35 on June 13, 2019 6.35 on July 1, 2019 30.48 on July 8, 2019 30.48 on July 15, 2019 0.4 on July 24, 2019 31.75 on July 29, 2019 31.75 on August 9, 2019	30.48 on July 2, 2019 30.48 on July 8, 2019 30.48 on July 15, 2019 30.48 on July 29, 2019	N/A
Total Rainfall (mm) (within growing season)	910.97 (764.92)	896.87 (752.86)	803.60 (655.90)

All three study sites grew maize (*Zea mays L.*) during the 2019 growing season and had fertilizer applied before each planting cycle, which usually occurs between late April and early May. Sites US-Ne1 and US-Ne2 were supplemented with water through a center-pivot irrigation system, while US-Ne3 relied solely on rainfall. Approximately one week before planting, nitrogen fertilizer was applied at the rate of 175 kg N ha^−1^ at US-Ne1, and 157 kg N ha^−1^ for US-Ne2 and US-Ne3, by coulter injection of liquid urea ammonium nitrate. In addition to this pre-plant application, a further 45 kg N ha^−1^ was applied at the irrigated sites at the beginning of July to improve maize N use efficiency^44^. Irrigation starts in mid-June and continues until early September, depending on weather, field conditions, and crop status^40^. A summary of each site is provided in Table 1.

### Planet CubeSat imagery

CubeSat data used in this study are sourced from Planet (www.planet.com), which operates a constellation of nano-satellites that achieve near-daily land coverage at 3 m spatial resolution. Each satellite is in a 3U configuration^28^ and is equipped with a multispectral camera that provides blue, green, red, and near-infrared (BGRN) reflectance bands. The Planet CubeSat constellation has inter-satellite differences that require correction for research purposes^30^. The CubeSat Spatio-Temporal Enhancement Method (CESTEM) was used to correct the CubeSat data and produce radiometrically calibrated surface reflectance datasets^28^. CESTEM is based on a machine-learning approach that performs multivariate regressions between high-quality reference imagery and the CubeSat bands using the Cubist 2.07 framework (RuleQuest; www.rulequest.com)^45^. The implementation of CESTEM employed here uses the HLS surface reflectance data set^46^ as the reference imagery. The HLS combines Landsat 8 and Sentinel-2 (A/B) imagery into a single harmonized data set that is Nadir Bidirectional Reflectance Distribution Function-Adjusted Reflectance (NBAR) at 30 m spatial resolution, and with the retrieval frequency of the combined satellite overpasses. Daily NBAR data from the Moderate Resolution Imaging Spectroradiometer (MODIS) Aqua and Terra satellites also feeds into CESTEM for calibration and gap-filling purposes to produce a daily cloud-free 3 m product from January 1 to October 31, 2019. The first step of the CESTEM methodology is to construct Planet Scope (PS) image stacks by intersecting available images to form a single mosaic. The mosaic process gives priority to PS images captured by newer CubeSats and to image strips captured by a single satellite to reduce cross-sensor inconsistencies. Next, the generated mosaics are calibrated against MODIS and resampled to a 30 m resolution after which an iterative cloud masking process is performed. The cloud-masked mosaics are then radiometrically harmonized using HLS reference data acquired over a ‘temporal calibration window’ around the capture time of the mosaic. In case there is no-coincident HLS-PS pair, HLS pixels are sampled from past/future day coincident pairs to produce a reference image that serves as the calibration reference (with preference given to HLS-PS pixels from closer to the date of interest and weighted as a function of surface reflectance change). Cubist is then used to produce regression relationships between Planet and the HLS reference. These Cubist regressions are then used to calibrate the original PS image. Finally, the harmonized PS-HLS imagery is gap-filled using a combination of cloud-free PS-HLS and MODIS pixels acquired both before and after (if possible) the given date, resulting in a cloud-free daily PS image that is HLS consistent. Further details on the original CESTEM methodology can be found in Houborg and McCabe^28^.

### Deriving an ultra-high resolution daily evaporation product

To produce the high spatiotemporal resolution maps of evaporation, the Priestley-Taylor Jet Propulsion Laboratory (PT-JPL) model^47^ is used in combination with Planet-CubeSat derived 3 m daily cloud-free NDVI data. PT-JPL has been evaluated across a wide range of scales and biomes^13,48,49^ and forms the modeling basis for producing land surface fluxes as part of NASA’s ECOSTRESS mission^20^. PT-JPL uses a small number of input parameters and partitions potential evaporation into three actual evaporation components (representing the soil (*LE*_*s*_), canopy (*LE*_*c*_), and interception (*LE*_*i*_) latent heat fluxes) using a set of biophysical constraints accounting for green canopy fraction $f_{g}$, plant moisture $f_{M}$, ambient temperature $f_{T}$, surface wetness $f_{wet}$, and soil moisture $f_{SM}$. The total latent heat flux is given by the sum of the three components i.e. $LE = LE_{s} + LE_{c} + LE_{i}$. Further details on the model can be found in Fisher, et al.^47^. We forced PT-JPL at hourly time intervals using CubeSat derived NDVI at 3 m resolution (assuming that NDVI stays constant throughout the day) together with meteorological information from each of the EC towers (only during the daytime). Spatially varying soil heat flux ($G$) was incorporated following the approach of Santanello and Friedl^50^. Instantaneous heat fluxes were evaluated against measured E at the field scale by extracting and averaging all the pixels contained within site boundaries. Flux estimates were converted to mm/h using:

1 $$ E\left( {mm/h} \right) = E\left( {W/m^{2} } \right)*3600/\lambda $$

where $\lambda$ is the latent heat of vaporization (approx. 2,260 kJ/kg). Values were then aggregated to mm/day to present the results in a water accounting context.

The performance of the retrieved E fluxes was evaluated using the coefficient of determination (r^2^) as a means to quantify the amount of the variability explained by the model, the mean bias (bias) that represents the over or under-estimation of the modelled fluxes, and the mean absolute error (MAE) as a measure of the average magnitude of the error disregarding direction. In contrast to the commonly used root mean squared error, the MAE gives equal weights to individual differences:

2 $$ r^{2} = \left( {\frac{{{\text{cov}} \left( {x,y} \right)}}{{\sigma_{y} \cdot \sigma_{x} }}} \right)^{2} $$

3 $$ bias = \frac{1}{n}\mathop \sum \limits_{i = 1}^{n} \left( {y_{i} - x_{i} } \right) $$

4 $$ MAE = \frac{1}{n}\mathop \sum \limits_{i = 1}^{n} \left| {y_{i} - x_{i} } \right| $$

## Results

### Monitoring heterogeneous field responses with CubeSat data

**Figure 2:**
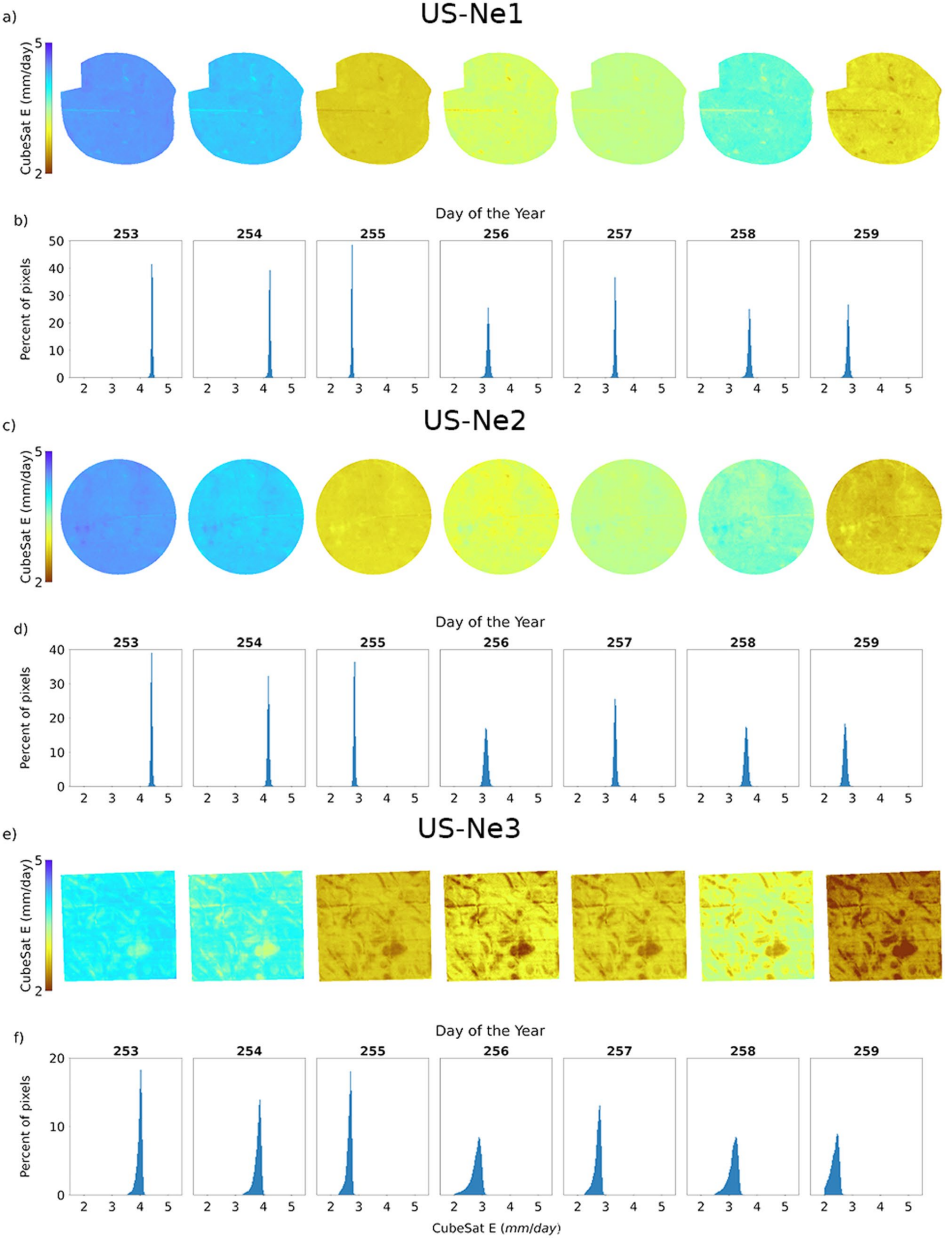
Spatial maps and related histograms of daily CubeSat-based E for the period September 9–15, 2019: approximately two months prior to harvest. Panels (**a**,**b**) correspond to US-Ne1, (**c**,**d**) to US-Ne2, and (**e**,**f**) to US-Ne3.

**Figure 3:**
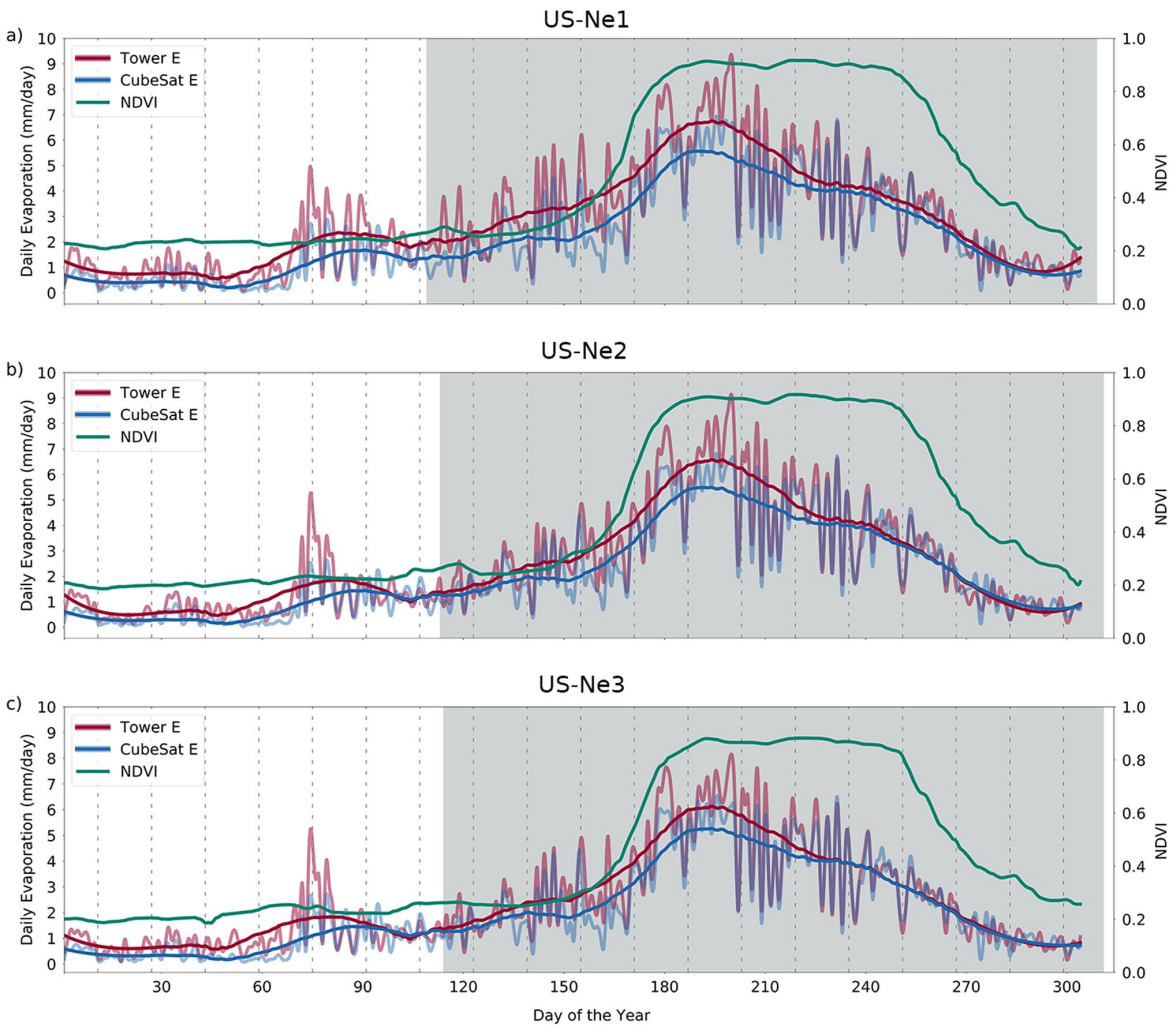
Time series of daily E for the CubeSat-based and closure-corrected EC measured fluxes, together with field-scale averaged NDVI for (**a**) US-Ne1, (**b**) US-Ne2, and (**c**) US-Ne3. The solid lines for the residual corrected and CubeSat E are smoothed versions of the original time-series. The shaded areas represent the length of the growing season (typically May–October), while the dashed lines represent the scheduled Landsat-8 overpasses.

Figure 2 presents a 7-day sequence of CubeSat-derived E retrievals for each of three studied fields, using both spatial maps and histograms to capture any underlying field-scale variability. The collection period corresponds to September 9–15, 2019 (day-of-year, DOY: 252-258), which is towards the end of the growing season. During this time, E presents a decreasing trend and a corresponding drop in NDVI (see Fig. 3). As can be seen from Fig. 2, the irrigated US-Ne1 and US-Ne2 sites show low variability in E, evidenced by the narrow distributions of their respective histograms (Fig. 2b,d), and presenting a maximum standard deviation of 0.06 and 0.07 mm/day, respectively. Even though both pivots are highly homogenous, small patches with different E can still be identified (Fig. 2a,c), which become more discernable towards the end of the week. For the rainfed US-Ne3 field, the spatial maps and the histograms (Fig. 2e,f) show higher variability (maximum standard deviation of 0.2 mm/day), reflecting the impact of no supplementary irrigation in this particular field. The higher variability areas on the spatial maps show lower E than their surroundings (Fig. 2e), which is also reflected in the wider histogram distributions (Fig. 2f).

A particular advantage of the CubeSat-driven simulations is that the data capture the development of the E fluxes throughout the course of the 2019 growing season on a day-to-day basis. Figure 3 shows the time-series of each field, produced based on the extracted field averaged E values (in mm equivalent). To highlight any changes in E development over time, a Savitzky-Golay smoothing filter^51^ was applied to each E time-series to remove temporal noise, while retaining the overall shape and trend^52^. In all cases, the CubeSat-based E follows similar dynamics to the measured E, with both being governed by the changing phenology of the crop (represented here by the time series of NDVI) and site meteorology. Given that NDVI remained stable during the season peak in July (around DOY 203), it can be inferred that E was controlled by the atmospheric evaporative demand and the available energy. Importantly, the CubeSat information enabled the discrimination of high-frequency day-to-day changes in E that would otherwise be missed if more traditional satellite platforms were used (i.e., Landsat-8). As Fig. 3 demonstrates, both the estimated and measured E have daily variations around the smoothed line, which could influence the total E amounts and lead to errors on irrigation scheduling when compared to coarser temporal resolution products. Indeed, the daily cloud-free product reveals a degree of variability in daily E between the scheduled Landsat-8 overpasses (represented by the dashed lines in Fig. 3). These changes in daily E likely reflect variability in available energy, which is driven principally by cloud cover diminishing the amount of solar radiation able to reach the crops. Even though the CubeSat derived NDVI did not present the same daily changes, the model was able to reproduce this variability as it was forced with on-site net radiation (Rn) measurements, which capture the influence of cloud cover.

**Figure 4:**
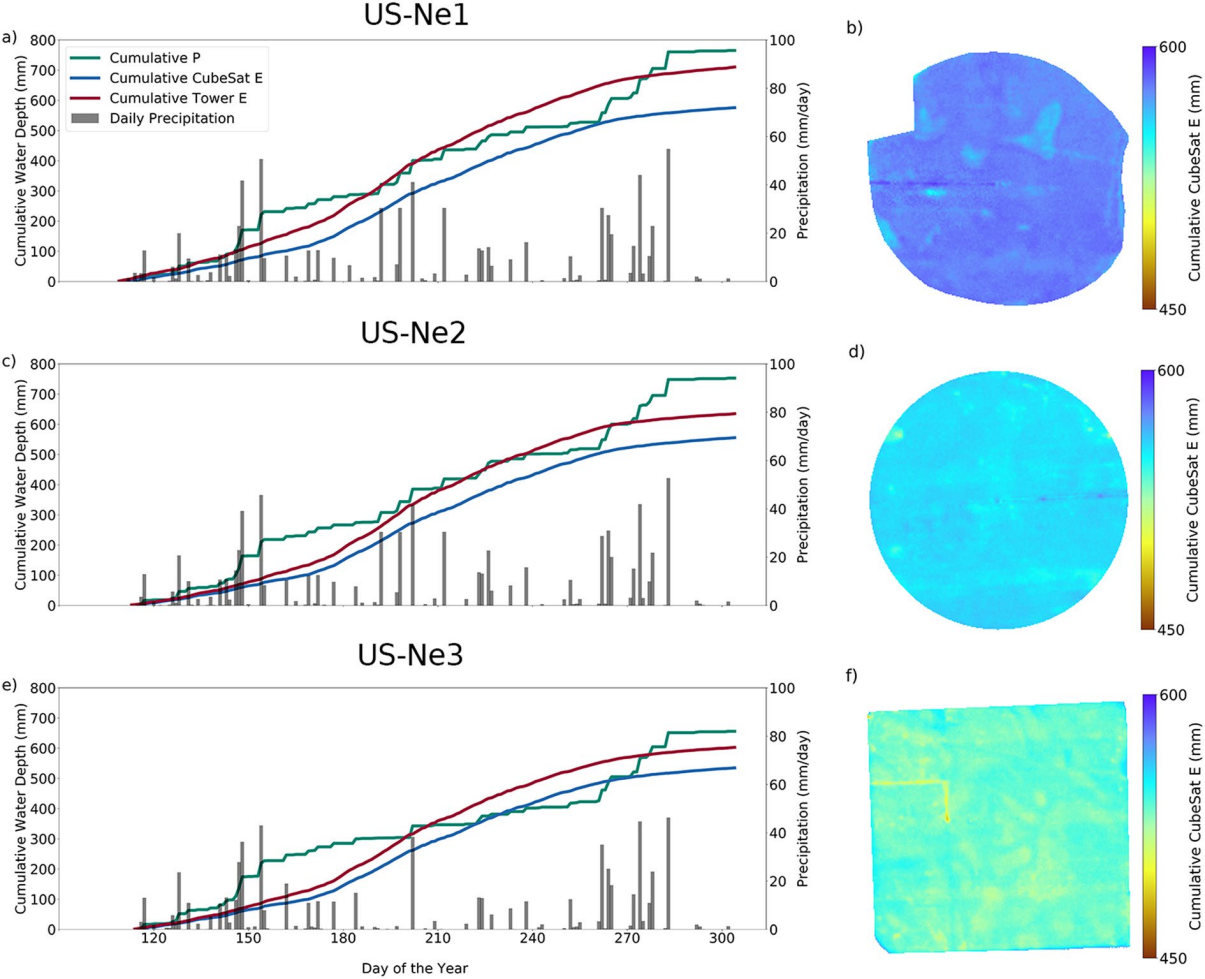
Cumulative plots of E and precipitation, together with spatial maps of cumulative E for the 2019 growing season (approximately DOY 109–311, see Table 1). The black bars represent the daily precipitation measured at each site. Panels (**a**,**b**) correspond to US-Ne1, (**c**,**d**) to US-Ne2, and (**e**,**f**) to US-Ne3.

To supplement the daily level E dynamics presented above, Fig. 4 compares the cumulative precipitation (mm) with the cumulative E (mm) for each field during the growing season. The cumulative CubeSat E estimated for the irrigated fields was lower than the precipitation amounts (Fig. 4a,c) which might indicate the lack of water stress conditions (assuming negligible runoff and infiltration to deeper soil layers), even if no irrigation was present to supplement the field. The CubeSat E underestimated the cumulative water depths compared to the cumulative E measured by the on-site tower by 18.98%, 12.51%, and 11.29% for US-Ne1, US-Ne2, and US-Ne3, respectively. It should be noted that this underestimation was accumulated throughout the season and that the tower E cannot show the spatial variability present in the CubeSat product. Importantly, these percentage errors are within the uncertainty levels of tower measurements^42^. Moreover, the estimated crop water use does not account for additional losses, such as percolation to deeper soil layers. The rain events for US-Ne1 and US-Ne2 are characterized by the same shape and magnitude (black bars in Fig. 4a,c), which is expected given their close proximity. Interestingly, the cumulative spatial variability for both fields (Fig. 4b,d) highlights areas of the field with lower E that cannot be appreciated in the spatial maps (panels a and c of Fig. 2). This field variability could point towards areas that were under-developed during early growth stages that subsequently improved as the season progressed. Since the irrigated fields received an additional water supply of 137.6 mm (US-Ne1) and 123.2 mm (US-Ne2), respectively from irrigation (see Table 1), the field variability may also reflect the underlying soil composition of the fields. Differences in nutrient amount and soil matrix structure have also been linked to crop yields at these sites^53^. On the other hand, the cumulative CubeSat E of the rainfed US-Ne3 site was higher than the cumulative precipitation amounts for the period August 19 until September 21 (35 days), which overlap with the sequence shown in the spatial maps of Fig. 2f. Given the rainfed conditions of US-Ne3, part of the spatial variability of the field could be linked to a change in soil moisture dynamics, leading to water stress^54^. On the other hand, the cumulative tower E was larger than cumulative precipitation for all sites. Interestingly, US-Ne3 had fewer precipitation events during the growing season than the other fields (Table 1), equivalent to a difference of 109 (US-Ne1) and 96 mm (US-Ne2) respectively, highlighting that even crops grown within a few kilometers of each other can experience quite different meteorological conditions.

Apart from providing crop water use insights, the high-resolution CubeSat-based retrievals facilitate the identification of underperforming areas, evidenced by low E values (and correspondingly low NDVI values). Given that areas with lower evaporation rates than their surroundings can be indicative of potential yield losses^55^, such information may be useful for prescribing remedial action in a timely manner. From a management perspective, the higher variability observed in US-Ne3 is likely the result of being rain-fed and serves as a useful contrast to the irrigated fields^56^. Being able to identify the within-field spatiotemporal variations in E provides a means for both farmers and water managers to allocate resources more effectively, driving precision agricultural improvements and optimizing end-of-season yields^57,58^.

### Performance of CubeSat-based evaporation retrievals

**Figure 5:**
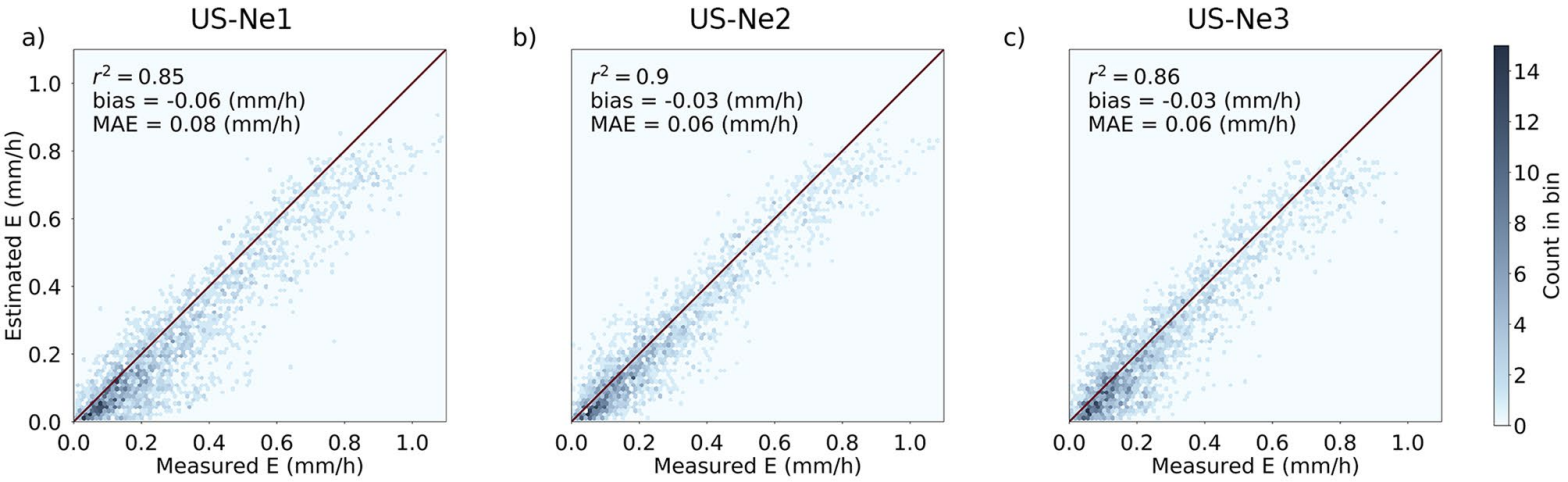
Scatter plots of CubeSat-based E against residual corrected eddy covariance E fluxes for each of the US-Ne1, US-Ne2, and US-Ne3 field sites.

While high spatiotemporal CubeSat retrievals are useful for identifying within field and daily variability, it is essential that crop water use estimates also have sufficient accuracy^32^ if they are to be of use for irrigation scheduling and other farm management activities. The gold-standard approach to evaluate E fluxes is by comparison against eddy covariance E flux measurements^40^. Figure 5 shows the evaluation of the E fluxes against hourly eddy covariance values from each study site during the growing season (from sowing to harvest, see Table 1). Across all sites, a strong correlation (r^2^ of 0.86, 0.9, and 0.86 for US-Ne1, US-Ne2, and US-Ne3, respectively) was observed between the measured and CubeSat-estimated fluxes. All sites presented negative bias values (normalized to the range of the measured values in parenthesis) of − 0.06 (5.51%), − 0.03 (2.76%), and − 0.03 (3.11%) mm/h for US-Ne1, US-Ne2, and US-Ne3, respectively. The consistent negative bias can be attributed to the particular E estimation model used herein, which has been shown to underestimate measured field E in a number of studies^12,17^. The main reason for the underestimation is that the model is sensitive to relative humidity^59^. More specifically, the modelling scheme assumes that the surface and atmospheric humidity are in equilibrium conditions, which is only the case for fully developed convective conditions^60^. A similar bias was reported in a recent study using the US-Ne1 and US-Ne2 sites^39^. It is also relevant to note that the majority of the fluxes were clustered around the lower end of the E range (i.e., from 0 to 0.25 mm/h), which was caused by an over-representation of the morning and afternoon fluxes in the satellite overpasses. Regardless, the relatively low bias, low mean absolute error, and the high correlation values (Fig. 5a–c) provide confidence in the accuracy of the estimated E fluxes to drive precision agriculture insights and decisions making.

## Discussion

Our study presents the first daily, cloud-free, 3 m resolution CubeSat-based crop water use product ever retrieved from space, and showcases its potential for advancing and delivering novel informatics that are of use to applications in precision agriculture. From these results, it is clear that CubeSat data provide an unprecedented opportunity to drive decision making in agricultural contexts, particularly when farmers are being urged to increase resource efficiency and to produce “more crop per drop”^61,62^. At the same time, those responsible for water resource allocation and management require information to fulfill their operational mandate: an area of particular relevance to state and federal governments that are responsible for water distribution systems and supplies^63^. Results illustrate the utility of high spatiotemporal resolution CubeSat imagery to deliver insights not just into water use, but also its temporal variability and spatial distribution.

In addition to the potential economic and productivity gains for farmers, a high spatiotemporal crop water use product is essential for ensuring more sustainable management of our food production systems. The Food and Agriculture Organization (FAO) estimates that by 2030 the world will require an additional 60% of food to feed an increasing global population^64^. What is less well appreciated is that this increased demand for food will require a commensurate demand for water. Unfortunately, many of our water systems are already under pressure to meet current food production goals^65^, so using irrigation more efficiently is necessary to both safeguard our supplies and meet food production objectives^66^. Beyond the food-water nexus, reducing irrigation rates also decreases on-farm energy costs, with more than 20% of the total energy consumption attributed to pumped water^67^. Knowledge of actual crop water use rates also enables the implementation of novel irrigation strategies such as deficit irrigation: a strategy that purposely allows the crops to undergo water stress outside critical crop-growth periods, with little impact to yield^68^ and offering improved water productivity and water use strategies^33^.

Although demonstrated via a relatively small scale case-study, the scalability of the approach is such that regional to global application is imminently achievable, offering a useful tool to inform and improve food and water security efforts^69^. Nevertheless, application beyond the field scale will benefit from the availability of higher spatial resolution meteorological data, which drive all E models^32^. While this might come from advanced numerical weather prediction schemes^70^, the increasing availability of distributed ground-based sensors in precision agriculture^71^ may be an alternative conduit for this much needed information. Furthermore, while reflectance derived vegetation indices are a good indicator of vegetation health and conditions, they cannot provide the early warning signals against yield losses relative to models that incorporate LST^72^ or fluorescence^73^. Unfortunately, such alternative sources of information do not currently provide the level of spatiotemporal resolution afforded by optical sensors. For instance, on an area-equivalent basis, the ECOSTRESS LST pixel (70 × 70 m; 4,900 m^2^) is around 5.5 times larger than the optical sensors from Landsat-8 (30 × 30 m; 900 m^2^), limiting the achievable spatial resolution of the ECOSTRESS evaporation product. Potential data fusion and downscaling approaches may provide a pathway to overcoming such constraints^21,74,75^.

It is worth noting that the daily cloud-free CubeSat data used in this study assumes that past observations can accurately recreate missing data (e.g., areas affected by cloud contamination). Indeed, depending on the duration of cloud-cover, gap-filling may not capture abrupt vegetation changes, such as field harvests^76^. One solution would be to explore multi-sensor fusion approaches, such as Sentinel-1 radar backscatter data, to act as an additional level of information on surface condition^77^. Additionally, imagery from the Planet CubeSat constellation requires radiometric correction and harmonization to avoid cross-sensor discrepancies, since the images collected over a given area are often taken by multiple CubeSats with inherent sensor differences^28,29^. The harmonization source for the CubeSat product used in this study derives from the harmonized Landsat and Sentinel-2 (HLS) surface reflectance data set^46^, which is dependent on the continuation of key satellite missions from the National Aeronautics and Space Administration (NASA) and the European Space Agency (ESA). One potential substitute for inter-satellite calibration could be the use of ground-based reflectance reference sites that can be used to perform vicarious calibration^78^. The feasibility of using ground targets would depend on the development of a calibration network, and would still be subject to clear sky conditions and satellite coverage of the calibration site(s)^79^.

Finally, while our study focused on crop water use, high-spatiotemporal CubeSat products can be of use in monitoring and forecasting other farm activities, such as crop health and development, either by themselves or through integration with crop biophysical models^80^. Indeed, improved approaches for yield prediction represents an incredibly valuable tool for both water and food security related issues^69^. One of the best approaches to predict crop development is by simulating crop growth based on weather and management information^81^. Recent studies have shown the considerable potential of using spatially distributed information from remote sensing to provide for enhanced prediction of yields^82^. Although advances have been made in the integration of remote sensing data and crop models, previous applications have tended to focus on coarser resolution satellite data, which inhibit any capacity to explore the intra-field variability required for in a precision agricultural context^83^. More generally, the daily CubeSat product developed herein can be of considerable benefit to applications beyond just agriculture, such as water resources management and security^84^, drought monitoring^85,86^ and ecosystem structure studies^87^ to name just a few. Future work should focus on exploring the various synergies afforded by these high spatiotemporal CubeSat datasets and their applications to support multidisciplinary insights.

## Data Availability

The CubeSat data that supports the findings of this study are available from Planet. Restrictions apply to the sharing of these images, which were used under license, and so are not available for redistribution. The in-situ meteorological and eddy covariance data used on this study is available at https://doi.org/10.17190/AMF/1246084, https://doi.org/10.17190/AMF/1246085, and https://doi.org/10.17190/AMF/1246086 for US-Ne1, US-Ne2, and US-Ne3 respectively.
